# Effects of Combined Nordic Hamstring and Speed Deceleration Training on Measures of Physical Fitness in Male Youth Soccer Players

**DOI:** 10.3390/jfmk11010084

**Published:** 2026-02-19

**Authors:** Yassine Negra, Senda Sammoud, Raja Bouguezzi, Younes Hachana, Helmi Chaabene

**Affiliations:** 1 Research Laboratory (LR23JS01) “Sport Performance, Health & Society”, Higher Institute of Sport and Physical Education of Ksar Saïd, University of “La Manouba”, Manouba 2037, Tunisia; yassinenegra@hotmail.fr (Y.N.); senda.sammoud@gmail.com (S.S.); rajabouguezzi@hotmail.com (R.B.); hachanayounes@gmail.com (Y.H.); 2 Higher Institute of Sport and Physical Education of Ksar-Said, University of Jendouba, Jendouba 8189, Tunisia; 3 Department of Cardiology and Angiology, University Hospital Magdeburg, 39120 Magdeburg, Germany

**Keywords:** children, team sports, eccentric training, athletic performance, individual response analysis

## Abstract

**Background:** A high level of physical fitness is a critical factor for optimal soccer performance. Therefore, developing key physical components such as sprinting, jumping, and change of direction (CoD) abilities from an early age is essential for both short- and long-term athletic success. While previous research in adolescent male athletes has demonstrated improvements in physical fitness following eccentric training, the effects of such training during the pubertal stage remain unclear. This study examined how an eccentric training program, combining the Nordic hamstring exercise with horizontal speed deceleration training, influenced physical fitness parameters in prepubertal soccer players. **Methods:** Thirty-six players were randomly divided into an ET group (n = 19) or an active control group (CG; n = 17). Both groups maintained their regular soccer training routines, with the ET group replacing 15 to 25 min of low-intensity drills with eccentric exercises twice per week for eight weeks. Pre- and post-intervention assessments included 20 m linear sprint speed, change of direction, agility, vertical jump, and standing long jump. **Results:** Significant group-by-time interactions were observed for all performance measures (*p* < 0.05), with moderate-to-large improvements in the ET group (*d* = 0.56 to 1.51; ∆3.83% to 14.95%) and no significant changes in the CG (*d* = 0.05 to 0.24; ∆0.38% to 1.31%). Individual response analysis indicated that 57 to 100% of players from the ET group and 23–58% from the CG group reached improvements beyond the smallest worthwhile change (SWC_0.2_). **Conclusions:** Collectively, these findings support the inclusion of eccentric training interventions, such as the Nordic hamstring exercise and horizontal speed deceleration training, to enhance sprinting, jumping, directional changes, and agility in young soccer players.

## 1. Introduction

Sprinting, jumping, change of direction (CoD) speed, and agility are among the key athletic qualities that significantly influence soccer performance [1]. These attributes are especially critical in youth soccer, where high-intensity actions often determine the outcome of decisive moments during a match [2,3]. It is, therefore, not surprising that elite level players typically display superior levels of muscular strength, speed, and their derivatives, including acceleration, sprint performance, CoD speed, and agility compared to their sub-elite counterparts [4]. While growth and maturation naturally contribute to improvements in these physical qualities among youth athletes [5], structured training interventions can further accelerate and enhance the improvements [6].

The integration of eccentric training (ET) in youth development programs has been endorsed in numerous studies. In fact, evidence indicates that ET improves multiple performance-related qualities, including muscle strength, jumping ability, sprint performance, and change-of-direction speed [7]. Research has shown that ET is more effective than concentric-only or combined concentric-eccentric methods for improving muscle power, hypertrophy, and performance in stretch-shortening cycle activities [8,9,10]. Among the most widely accessible eccentric exercises is the Nordic hamstring exercise (NHE) [11]. Recently, another effective eccentric exercise targeting the knee extensors has emerged; the horizontal speed deceleration training (HSDT) [12]. Both the NHE and HSDT have received growing attention over the last years due to their efficacy in improving various measures of physical fitness across different individual and team sports [12,13,14]. For instance, Negra et al. [12] revealed that an in-season HSDT executed alongside regular handball-specific training resulted in moderate to large improvements on measures of CoD, jumping ability, and repeated sprint ability outcomes (∆2.49 to 16.25%; *d* = 1.01 to 1.72) performance in youth male handball players aged 15 years. Similarly, Bouguezzi et al. [14] showed that an 8-week NHE program resulted in meaningful gains in CoD [505 test] (7.36%; *d* = 0.92), agility (y-shaped test) (∆7.91%; *d* = 1.68), and jumping ability (CMJ: ∆7.44%; *d* = 0.35), in pubertal male soccer players (age = 14.8 ± 0.2 years).

While previous studies have explored the effects of NHE on athletic performance [12,13] and to lesser extent the effects of HSDT [12], the combined effects of both have never been investigated in youth soccer players. Given their complementary targeting of both knee flexors and extensors in eccentric actions, combining these two methods could offer synergistic benefits for developing lower-limb power and neuromuscular control. Additionally, the majority of existing research has focused on adolescent or adult athletes, with relatively few intervention studies targeting prepubertal soccer players, particularly eccentric training. Youth athletes in the prepubertal stage exhibit unique developmental profiles that demand age-specific training approaches. This developmental window is also considered critical for motor learning and long-term athletic development, making it an ideal period to introduce evidence-based interventions aimed at enhancing both performance and injury resilience. Therefore, the present study aimed to investigate the effects of an 8-week ET program combining the NHE and HSDT on sprinting, jumping, CoD speed, and agility in prepubertal male soccer players. We hypothesized that integrating an ET program combining the NHE and HSDT into a traditional soccer training regimen would lead to larger improvements in measures of physical fitness compared to standard soccer training alone [13,14].

## 2. Methods

### 2.1. Experimental Approach to the Problem

**Figure 1:**
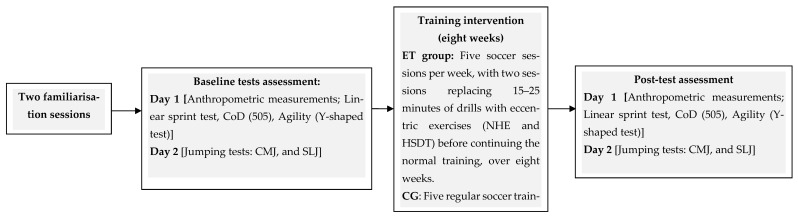
Schematic presentation of the experimental design. ET: eccentric training; CG: control group, CoD: change of direction; CMJ: countermovement jump; SLJ; standing long jump; NHE: Nordic hamstring exercise; HSDT: horizontal speed deceleration training.

This randomized controlled trial examined the effects of an ET program, focusing on the NHE and HSDT, on measures of physical fitness in prepubertal male soccer players (Figure 1). Thirty-eight prepubertal male soccer players were randomly assigned to either an eccentric training (ET) group or an active control group (CG) using a computer-generated randomization sequence (https://www.random.org/lists/, accessed on 12 March 2025). Both groups participated in five soccer training sessions per week. The ET group replaced 15 to 25 min of low-intensity soccer drills with eccentric exercises on Tuesdays and Fridays, while continuing their regular soccer-specific training afterward. In contrast, their counterparts maintained their regular in-season soccer-specific training regimen. One week before baseline testing, two familiarization sessions were conducted to help participants acclimate to the testing procedures. A variety of physical fitness tests were used to assess changes before and after the ET program, including 20 m linear sprint speed, CoD (505 CoD test), agility (Y-shaped agility test), jump height (countermovement jump [CMJ]), and jump distance (standing long jump [SLJ]). Physical fitness tests were performed in a fixed order over three consecutive days (Figure 1). All tests were scheduled at least 48 h after the last training session or match and were conducted at the same time of day (17:00–18:30).

### 2.2. Participants

**Figure 2:**
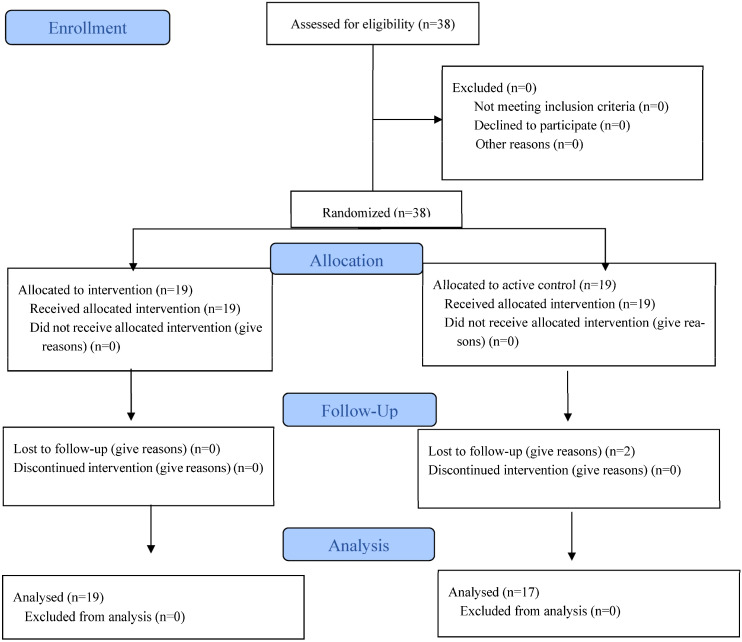
The CONSORT diagram includes detailed information on the interventions received.

Figure 2 displays a Consolidated Standards of Reporting Trials (CONSORT) diagram of the levels of reporting and participant flow for the study. With reference to a recent similar study by Bouguezzi et al. [14], an a priori power analysis, with a type I error rate of 0.05 and 90% statistical power was computed. The analysis indicated that a total of 20 participants would be sufficient to observe a significant interaction effect (effect size Cohen’s *d* = 0.80 for the 505 CoD best). To account for potential participant attrition, a total of 38 soccer players from two different regional soccer teams were allocated to either the ET group (n = 19) or the active CG (n = 19). Two participants from the CG were lost to follow-up, leaving 17 participants included in the analysis. All participants were competitive male soccer players, with an average of 4.0 ± 1.2 years of systematic soccer training, competing at the regional level within the national soccer federation. Players were registered with their respective clubs and regularly participated in official league matches and regional competitions during the competitive season. All participants were healthy and had not experienced any musculotendinous injuries during the six months prior to the start of the study. The anthropometric characteristics of both groups are presented in Table 1. Biological maturity was estimated using the maturity offset (MO) approach described by Moore et al. [15]. The following predictive equation was applied: MO = −7.999994 + (0.0036124*age*height).

### 2.3. Linear Sprint Test

The twenty-meter linear sprint performance was measured using a single-beam electronic timing system (Microgate SRL, Bolzano, Italy). Participants began from a standing split-stance position, with the lead foot placed 0.3 m behind the first infrared photoelectric gate. This gate was positioned 0.75 m above the ground to detect trunk movement and minimize false triggering caused by limb motion. Two single-beam photoelectric gates were used for timing. Participants were instructed to avoid any rocking movements or false steps prior to the start. The between-trial recovery time was three minutes. The best performance out of two trials was used for further analysis. The intra-class correlation coefficient (ICC) for between-trial reliability was 0.94.

### 2.4. The 505 Change of Direction Test

The 505 CoD test was conducted according to the previously published procedures [12], using an electronic timing system (Micro gate, Bolzano, Italy). Participants adopted a standing split-stance position 10 m from the start/finish line, sprinted maximally through the timing gates, executed a 180° turn at the 15 m line marked by a cone, and then returned as quickly as possible through the start/finish line. To ensure correct test execution, a researcher was positioned at the turning point; trials were invalidated if players changed direction before reaching the marker or pivoted on the incorrect foot and were repeated following the designated recovery period. A between-trial rest period of three minutes was provided. The best performance out of two trials was used for further analysis. The ICC for between-trial reliability was 0.90.

### 2.5. Y-Shaped Agility Test

Four pairs of electronic timing gates (Micro gate, Bolzano, Italy) were set up for the test. Participants started each trial 20 cm in front of the starting line and sprinted the first 5 m at maximum speed toward the second timing gate, which then indicated the direction of the subsequent movement. Upon crossing the middle gate, the next gate was activated, requiring participants to change direction as quickly as possible while maintaining maximal speed over the final 10 m [17]. Each participant performed two trials, running either to the left or right in a randomized order after the initial 5 m. The fastest time of the two trials was used for analysis. The ICC for between-trial reliability was 0.82.

### 2.6. Countermovement Jump Test

For this test, participants began from an upright standing position and executed a rapid downward movement by flexing the knees and hips, immediately followed by a maximal vertical jump through full leg extension. Throughout the jump, participants were instructed to keep their hands on their hips (arms akimbo). The jump height was measured using a floor-level optoelectric system (Optojump, Microgate SRL, Bolzano, Italy). A one-minute rest interval was provided between trials, and the highest jump out of three attempts was used for subsequent analysis. The ICC for between-trial reliability was 0.88.

### 2.7. Standing Long Jump Test

During the bilateral standing long jump (SLJ) test, participants stood with their feet shoulder-width apart, toes positioned behind the starting line. Upon the command “ready, set, go,” they performed a rapid leg flexion and downward arm swing before jumping as far forward as possible. Participants were instructed to land with both feet simultaneously and to avoid falling forward or backward. The horizontal distance from the starting line to the heel of the rear foot at landing was measured with a tape measure to the nearest centimeter. A one-minute rest period was provided between trials, and the longest jump of two attempts was used for analysis. The ICC for between-trial reliability was 0.84.

### 2.8. Eccentric Training Program

All details related to the eccentric training (ET) intervention are provided in Table 2. The ET program was implemented during the first half of the in-season period (November–December 2024) and lasted 8 weeks with two sessions per week. The intervention consisted of two eccentric focused-exercises: the Nordic hamstring exercise (NHE) and the horizontal speed deceleration exercise. Each ET session was preceded by a standardized warm-up including 2–3 min of jogging and dynamic movements (e.g., high knees, butt kicks), followed by 2–3 min of dynamic stretching (e.g., leg swings, arm circles, hip openers). The weekly training schedule included two ET sessions: the first session took place on Tuesdays, at least 48 h after the last match played over the weekend, while the second session was conducted on Fridays, 72 h after the first session. For the NHE, participants focused on a slow controlled descent with progressively reduced partner hand assistance. During weeks 1–2, partial assistance was provided by having the partner kneel in front of the participant and support the participant’s torso with both hands placed at the shoulders. In weeks 3–4, the participants gradually reduced manual support, providing assistance only when necessary to maintain controlled movement. During weeks 5–6, minimal hand assistance was given, limited to slight support near the end range of motion to allow maximal eccentric loading. By weeks 7–8, participants performed the exercise independently, maintaining full control throughout the entire movement. A 10-s and 90 s rest interval was provided between repetitions and sets, respectively. For the HSDT, each repetition consisted of a 20 m maximal acceleration followed by a 5 m deceleration, with participants instructed to (i) take shorter controlled steps, (ii) maintain an upright posture with a slight forward lean during deceleration, and (iii) keep the center of gravity low with slightly bent knees to absorb impact. Ninety-second and 3 min rest intervals were allocated between repetitions and sets, respectively. The total running distance (acceleration + deceleration) per week gradually increased from 300 m during the first week to 500 m during the last training week.

This program was specifically adapted for prepubertal male soccer players to support neuromuscular development and reduce injury risk through age-appropriate eccentric loading strategies.

### 2.9. Statistical Analyses

All data analyses were performed using SPSS version 25.0 (SPSS, Inc., Chicago, IL, USA). Data are presented as the means and standard deviations (SD). The normality assumption was tested and confirmed using the Shapiro–Wilk test. To determine the effect of the interventions on the dependent variables, a 2 (group: ET and CG) × 2 (time: pre, post) ANOVA with repeated measures was conducted for each parameter. When group × time interactions reached the level of significance (i.e., significant F value), group-specific post hoc tests (i.e., paired *t*-tests) were used. To determine the magnitude of the training effect within and between groups, Cohen’s *d* effect size (d) was calculated. According to Hopkins et al. [18], *d* values are classified as trivial (<0.2), small (0.2–0.6), moderate (0.6–1.2), large (1.2–2.0), very large (2.0–4.0), and extremely large (>4.0). Test–retest reliability was assessed using the ICC. The SWC represents the smallest change in a performance variable that is considered practically meaningful. It helps to determine whether observed changes are likely to have real-world relevance, regardless of statistical significance [19]. In athletes, the SWC is typically calculated as 0.2 multiplied by the between-subject standard deviation of the particular test [20]. The calculation is based on Cohen’s effect size principle, with 0.2 representing a small, but not trivial, effect size. The alpha level of significance was set at *p* < 0.05.

## 3. Results

All participants were at the prepubertal stage, with an equal distribution between the two groups. Both groups demonstrated a high level of compliance with the intervention. All participants who completed the study attended 100% of the prescribed training sessions, and no exclusions due to insufficient attendance were required. Two participants from the control group withdrew during the intervention period for personal reasons unrelated to the study protocol. Throughout the intervention period, no training- or testing-related injuries were reported in either group, indicating good tolerability of the ET program. Furthermore, the competitive match exposure was comparable between groups, with no significant differences observed in total match minutes played during the intervention period (*p* > 0.05). Over the 8-week intervention, the cumulative match duration was approximately 490–560 min per participant in each group, reflecting similar on-field exposure. Physical fitness outcomes were assessed at both baseline and post-intervention, with the results detailed in Table 3. At baseline, there were no significant differences between the groups in terms of anthropometric data or physical fitness levels.

### 3.1. Linear Sprint Speed

Our results revealed a significant main effect of time for the 20 m sprint (*d* = 0.87 [moderate]; *p* < 0.05), along with a significant group × time interaction (*d* = 1.18 [moderate]; *p* < 0.01). Post hoc comparisons indicated a moderate improvement in sprint performance from pre- to post-intervention in the eccentric training group (Δ 3.83%; *d* = 0.68; *p* < 0.01), whereas the control group showed no significant change (Δ 0.62%; *d* = 0.11 [trivial]; *p* > 0.05). Individual response analysis further revealed that the ET group had the highest proportion of participants exceeding the SWC_0.2_ threshold (68%; n = 13), compared with 29% (n = 5) in the CG.

### 3.2. Change of Direction Speed

For the 505 CoD speed test, there was a significant main effect of time (*d* = 1.32 [large]; *p* < 0.01) and a significant group × time interaction (*d* = 0.77 [moderate]; *p* < 0.05). Post hoc analysis indicated a moderate improvement in 505 CoD speed from pre- to post-intervention for the eccentric training group (Δ 5.39%; *d* = 0.84; *p* < 0.01), whereas the control group exhibited no significant changes (Δ 0.38%; *d* = 0.00 [small]; *p* > 0.05). Individual response analysis showed that 84% of the ET group (n = 16) and 58% of the control group (n = 10) achieved improvements exceeding the SWC_0.2_ threshold.

### 3.3. Y-Shaped Agility Test

Our results revealed a significant main effect of time (*d* = 1.07 [moderate]; *p* < 0.01) and a significant group × time interaction (*d* = 0.74 [moderate]; *p* < 0.05) for the Y-shaped agility test. Post hoc analyses indicated a moderate improvement from pre- to post-training in the eccentric training group (Δ 5.34%; *d* = 0.80; *p* < 0.05), while the control group showed no significant changes (Δ 0.94%; *d* = 0.24 [small]; *p* > 0.05). Individual response analysis revealed that 63% of the ET group (n = 12) and 52% of the CG (n = 9) achieved improvements exceeding the SWC_0.2_ threshold.

### 3.4. Vertical Jumping

For the CMJ height, there was a significant main effect of time (*d* = 0.67 [moderate]; *p* < 0.05) and a significant group × time interaction (*d* = 0.80 [moderate]; *p* < 0.05). Post hoc analyses revealed a small pre-to-post improvement in CMJ height for the ET group (Δ 9.29%; *d* = 0.56 [small]; *p* < 0.05), while the CG showed no significant change (Δ 0.90%; *d* = 0.05 [trivial]; *p* > 0.05). Individual response analysis indicated that 57% of the ET group (n = 11) and 23% of the CG (n = 4) achieved improvements exceeding the SWC_0.2_ threshold.

### 3.5. Horizontal Jumping

For the SLJ test, there was a significant main effect of time (*d* = 1.82 [large]; *p* < 0.001) and a significant group × time interaction (*d* = 2.19 [very large]; *p* < 0.001). Post hoc analysis revealed a significant pre-to-post improvement in the ET group (Δ 14.95%; *d* = 1.51 [large]; *p* < 0.001), whereas the CG showed no meaningful change (Δ 1.32%; *d* = 0.11 [trivial]; *p* > 0.05). Individual response analysis indicated that all participants in the ET group (100%; n = 19) exceeded the SWC_0.2_ threshold, compared with only 23% (n = 4) of the CG.

## 4. Discussion

This investigation sought to evaluate the impact of an ET program incorporating the NHE and HSDT on physical fitness parameters in prepubertal male soccer players. The findings indicated that partially replacing regular soccer training with eccentric-based exercises resulted in small to large improvements across all the assessed fitness measures. In contrast, players who followed their normal soccer training showed no significant physical fitness enhancements, highlighting the value of incorporating specific eccentric exercises into conditioning programs.

### 4.1. Linear Sprint Performance

Sprinting ability, particularly rapid acceleration over short distances, is a crucial component of soccer performance, as it frequently precedes decisive moments such as goal-scoring opportunities, critical passes, or defensive recoveries during high-intensity phases of the game [21,22]. Our study revealed that the ET intervention generated moderate improvements in 20 m sprint performance (*d* = 0.68), while regular soccer training resulted in no meaningful changes. Sprint gains are likely mediated primarily by neural adaptations rather than hypertrophy, due to low circulating testosterone [23]. Additionally, individual response analysis showed that 68% of participants in the eccentric training group (n = 13) and only 29% of those in the control group (n = 15) improved their sprint performance beyond the SWC_0.2_. These results align with the previous literature, supporting the efficacy of ET in improving sprint performance in youth athletes. Recently, Bouguezzi et al. [14] contrasted the effects of 1 versus 2 days per week volume-equated ET using the NHE and reverse Nordic exercise (RNE) on physical fitness in youth male soccer players. Their results indicated that spreading ET over two weekly sessions produced superior improvements in sprint speed compared to a single weekly session. Similarly, Sammoud et al. [24], revealed a moderate pre-to-post performance improvement for the 10 m (∆-2.24%; ES = −0.67]) and 20 m (∆-3.24%; ES = 0.75) linear sprint performance following an 8-week program of combined Nordic and reverse Nordic hamstring exercises in prepuberal male soccer players. Abdelkader et al. [13] found smaller but meaningful gains (*d* = 0.42) in 30 m sprint times following an eight-week NHE training in prepuberal male soccer players. More recently, Negra et al. [25] revealed a large improvement (∆3.81%; ES = 2.03) in 20 m sprint performance in youth tennis players following a 6-week HSDT training, while in the active CG, no significant change was detected. Of note, no measures of the potential mechanistic underpinnings were conducted in this study. While speculative, the sprint performance gains following eccentric training may be primarily associated with neural adaptations, including heightened neural drive to the active musculature [8]. These adaptations may have led to improvements in muscle strength and power, ultimately enhancing the sprint speed.

### 4.2. Change of Direction and Agility

Change of direction speed and agility are essential qualities in modern soccer, due to the sport’s dynamic multidirectional nature. In the current study, the ET group revealed moderate improvements in CoD speed and agility (*d* = 0.84 and 0.80, respectively), highlighting the effectiveness of the adopted ET interventions. These improvements are likely driven by neural adaptations, including enhanced motor unit recruitment and intermuscular coordination, rather than muscle hypertrophy [23]. Individual response analysis indicated a higher proportion of youth players (84% and 63%; n = 16 and 12, for CoD speed and agility, respectively) from the ET group who improved their CoD speed and agility performance to a level that exceeded the SWC_0.2_. In the CG, the results indicated that only 58% (n = 10) of participants showed improvements above the same threshold for CoD speed and 52% (n = 9) for agility. These results align with the findings of Negra et al. [12], who reported large gains in the CoD performance (*d* = 1.72) following an 8-week HSDT program in youth handball players. For instance, Bouguezzi et al. [14] observed that distributing ET across two sessions per week resulted in larger enhancements in CoD speed than a single weekly session, with male youth soccer players demonstrating a moderate performance gain (*d* = 0.92) after completing an 8-week program combining the RNE and NHE. More recently, Sammoud et al. [24], revealed a moderate improvement in 505 CoD times from pre to post (∆-4.87%; *d* = 0.98) following an 8-week combined NHE and RNE training in prepubertal male soccer players. Following a 6-week HSDT program, Negra et al. [25] demonstrated large performance improvement in the 505 CoD (∆–6.02%; ES = 1.62), and agility (∆–6.28%; ES = 1.14) in pubertal tennis players. The performance gains observed in our study likely stem from the well-documented impact of ET on knee extensor and flexor strength [26,27], which plays a pivotal role in the deceleration phase of CoD tasks [28,29]. Enhanced eccentric capacity optimizes braking during rapid deceleration, allowing athletes to better control momentum and reaccelerate efficiently in the new direction [30]. Moreover, eccentric strength contributes to joint stability and more efficient force transmission across joints, which improves motor control and may reduce injury risk. These neuromuscular and biomechanical adaptations collectively support the improved CoD and agility performance observed in this study.

### 4.3. Jumping Ability

Jumping ability is a critical skill in soccer, especially for actions such as heading, winning aerial duels, and recovering high balls in both offensive and defensive phases. These actions demand high vertical and horizontal jump performance. In our study, the ET program led to small to large improvements in CMJ height and SLJ distance (*d* = 0.56 and 1.51, respectively, for the CMJ, and SLJ test). In contrast, the CG showed no significant improvements in either CMJ or SLJ performance. In confirmation of these findings, 57% (n = 11) of participants in the ET group exceeded the SWC_0.2_ for vertical jump performance, whereas only 41% (n = 7) of the active control group reached this threshold. Similarly, while 100% of the ET group improved their SLJ distance beyond the SWC_0.2_ threshold, only 23% (n = 4) of the CG reached the same level of improvement. These findings corroborate earlier investigations that demonstrate the effectiveness of ET in youth. Negra et al. [12] reported a substantial gain in CMJ performance (*d* = 1.70) after an eight-week HSDT program in prepuberal handball players. Similarly, Abdelkader et al. [13] observed moderate (*d* = 0.84) and large (*d* = 1.39) gains in SLJ distance in prepubertal male soccer players after one or two weekly NHE sessions, respectively. More recently, Negra et al. [25] reported large improvement in CMJ (∆8.00%; ES = 1.29) and SLJ (∆7.80%; ES = 1.13) following a 6-week HSDT program in prepubertal tennis players. Supporting this evidence, a meta-analysis by Maroto-Izquierdo et al. [31] showed that eccentric overload training with flywheel devices led to significantly larger improvements in vertical jump performance (*d* = 0.46) compared to the control groups. While the exact mechanisms remain under debate, these improvements are likely mediated by neuromuscular adaptations, including enhanced motor unit recruitment and increased firing frequency, especially during short-term interventions. [32,33].

### 4.4. Limitations

Several limitations of this study should be considered. First, only performance measures were evaluated, with no evaluation of the potential underlying physiological mechanisms (e.g., changes in muscle architecture, tendon stiffness, or neuromuscular adaptations). Incorporating objective physiological (e.g., blood biomarkers) or imaging-based (e.g., ultrasound) measures in future research would help clarify how ET drives these performance gains. Second, the intervention period was relatively short, restricting conclusions to its specific duration. As a result, it is not possible to infer the effects of longer-term eccentric training, which should be addressed in future research. Third, the absence of assessor blinding during performance testing may have caused measurement bias, affecting the objectivity of the results. Upcoming investigations should consider blinding the assessors to reduce any measurement bias risk.

## 5. Conclusions

The results of this study demonstrate that adding a short-duration eccentric training program (15–25 min per session, twice per week), including NHE and HSDT, to the normal training routine of prepubertal male soccer players can meaningfully enhance various measures of physical fitness. After eight weeks, the participants showed moderate-to-large gains in linear sprinting ability, agility, CoD speed, and vertical as well as horizontal jumping capacity. In contrast, players who maintained their usual soccer training alone showed no substantial improvements in these fitness variables. From a practical perspective, pediatric strength and conditioning coaches may consider integrating NHE and HSDT exercises into regular soccer training routine, as these exercises are time-efficient, require no-to-minimal equipment, and can easily be implemented within warm-up or conditioning phases of the session. Further research involving larger cohorts is recommended to confirm these outcomes and to strengthen the current evidence.

## Figures and Tables

**Table 1:** Anthropometric characteristics of the included participants.

	ET Group (n = 19)	CG (n = 17)	Independent Sample *t*-Test (95%CI)	*p* Value
Age (years)	12.50 ± 0.24	12.40 ± 0.22	1.21 (−0.06 to 0.26)	*p* > 0.05
Body height (cm)	148.63 ± 6.55	152.98 ± 6.47	−1.92 (−8.98 to 0.27)	*p* > 0.05
Body mass (kg)	40.28 ± 5.28	40.60 ± 4.96	−0.17 (−3.94 to 3.31)	*p* > 0.05
Maturity Offset (years)	−1.29 ± 0.32	−1.15 ± 0.35	−1.24 (−0.38 to 0.09)	*p* > 0.05

Notes: Data are presented as means and standard deviations; ET = eccentric training group; CG = control group. All experimental procedures and potential risks were clearly explained prior to participation. Written informed consent was obtained from parents or legal guardians, and assent was provided by the participants before the study began. The research protocol received approval from the local Institutional Review Committee of the Higher Institute of Sport and Physical Education, Ksar Said, Tunisia (Approval No. LR1501-203) and was carried out in accordance with the most recent version of the Declaration of Helsinki [16].

**Table 2:** Eccentric training program.

Week	Mode	Sets	Repetitions	Rest (s)	Mode	Sets	Acceleration Distance	Deceleration Distance	Repetitions	Rest (s)
1	NHE	2	6	90	HSDT	2	20	5	6	90
2	NHE	2	6	90	HSDT	2	20	5	6	90
3	NHE	3	8	90	HSDT	2	20	5	8	90
4	NHE	3	8	90	HSDT	2	20	5	8	90
5	NHE	3	10	90	HSDT	2	20	5	10	90
6	NHE	4	10	90	HSDT	2	20	5	10	90
7	NHE	4	10	90	HSDT	2	20	5	10	90
8	NHE	4	10	90	HSDT	2	20	5	10	90

HSDT: Horizontal speed deceleration training; NHE: Nordic hamstring exercise.

**Table 3:** Evolution of group-specific physical performance from the beginning to the end of the study.

	ET (n = 19)				CG (n = 17)				ANOVA	
	Pretest		Posttest		Pretest		Posttest		*p*-Value (d)	
	M	SD	M	SD	M	SD	M	SD	Time	Group × Time
Linear sprint speed test										
20 m sprint (s)	3.85	0.20	3.71	0.22	3.61	0.15	3.63	0.20	<0.05 (0.87)	<0.01 (1.18)
Change of direction speed test										
505 CoD speed test (s)	2.93	0.20	2.77	0.19	2.66	0.20	2.62	0.20	<0.01 (1.32)	<0.05 (0.77)
Agility										
Y-shaped test (s)	3.03	0.22	2.87	0.19	3.06	0.16	3.03	0.12	<0.01 (1.07)	<0.05 (0.74)
Muscle power										
CMJ (cm)	23.39	3.70	25.57	4.25	21.80	4.20	21.60	4.03	<0.05 (0.67)	<0.05 (0.8)
SLJ (m)	1.59	0.13	1.82	0.18	1.66	0.16	1.64	0.20	<0.001 (1.82)	<0.001 (2.19)

M: means; SD: standard deviation; ET: eccentric training group; CG: control group; d: Cohen’s d; CoD: change of direction; CMJ: countermovement jump; SLJ: standing long jump.

## Data Availability

The original contributions presented in this study are included in the article/Supplementary Material. Further inquiries can be directed to the corresponding author.

## Supplementary Materials

The following supporting information can be downloaded at: https://www.mdpi.com/article/10.3390/jfmk11010084/s1.
